# Global Health as “umbrella term” – a qualitative study among Global Health teachers in German medical education

**DOI:** 10.1186/s12992-018-0352-y

**Published:** 2018-03-27

**Authors:** Matthias Havemann, Stefan Bösner

**Affiliations:** 1 Department of Family Medicine, Philipps-University Marburg, Karl-von-Fritsch-Straße 4, D-35043 Marburg, Germany; 2 London School of Hygiene and Tropical Medicine, Keppel Street, London, WC1E 7HT UK

**Keywords:** Global Health, Medical education, Globalisation, Qualitative research, Grounded theory

## Abstract

**Background:**

The increasing impact of globalisation on healthcare demands new knowledge, skills and attitudes from healthcare professionals. One consequence of this is the rise of Global Health (GH) programs in health education all over the world. In Germany no consensus exists on what GH is and how it should be taught. This study used a grounded theory approach. We conducted eleven in-depth interviews with GH teachers in German medical education to ask them about their views on Global Health and the approaches they took in teaching these topics. Data collection and Analysis informed each other and followed an inductive approach.

**Results:**

Our research identified three major questions: (1) What is GH? (2) What belongs to GH? (3) How can GH be taught? A central finding of our study is the understanding of GH as an umbrella term. We show how this understanding helps clarify the relation between GH and Public Health, International Health and Tropical medicine. At the core of GH we see the supraterritorial determinants of health. Surrounding the core, we describe a wide variety of topics that are a facultative, but not necessarily a compulsory part GH. One of the key characteristics of GH within all its aspects is its multidisciplinary nature. Based on this understanding we present models about the content of GH, how it can be taught and how GH teaching improves and strengthens overall medical education.

**Conclusions:**

This is to our knowledge the first study that conducts in-depth interviews with GH teachers to explore the practical understanding of GH in medical education. While the generalisability of our results needs to be subject of further research, the models presented can help shape the future discourse around GH and its integration into medical education.

**Electronic supplementary material:**

The online version of this article (10.1186/s12992-018-0352-y) contains supplementary material, which is available to authorized users.

## Background

### Globalisation and its impact on health

In recent years the phenomenon of globalisation has received increased attention in healthcare research and praxis [1]. Various academic disciplines have extensively written on globalisation’s impact on health. Globalization can be best understood as the “*rise of transplanetary connectivity*” [2]: Developments in telecommunications, technology, transport and international systems have changed the way we communicate and interact. While many of these interactions remain within countries, nations or regions, the transplanetary and supraterritorial aspects have gained a stronger influence. These phenomena are not completely new, but the extent of supraterritorial connectivity with its global instantaneity and simultaneity is unprecedented so far and demands new perspectives and research methods to be fully understood [3]. Supraterritoriality includes and extends the concept of supranationality: it reflects the diverse nature of global actors and their relation to classical actors that were bound to national boundaries and thereby includes countries, associations of countries and communities or institutions that span across several countries without necessarily fully including them.

Globalisation has created a new social space. The reduction of barriers strengthens connections between people all over the world [3]. This globality can be found in various fields: e.g. communications and media, travel and migration, organisations, transnational companies, consumption habits, military activities, ecology, law and in a social, global consciousness [3]. This new global social space does not devalue or replace but even fosters locality and regionality, because these are the spaces in which its effects take place [3].

Various attempts have been made to draw a framework of the impact of globalisation on health [4, 5]. Actors in academic global health call for an interdisciplinary systems approach [6, 7] and integrative thinking to understand the complex effects and relationships in global health [8, 9].

But, next to the implications for research and governance, globalisation has also practical ramifications in physicians’ day-to-day work. Increased “cross-border flows of goods, services, capital, people, information and ideas” [10] challenge clinical routines. Intercultural and linguistic barriers in treating migrants, healthcare worker migration, increased risk of global pandemics, simplified access to global knowledge or complex international intellectual property law demand new skills, knowledge and attitudes from healthcare professionals.

The Lancet commission “Education of Health professionals for the 21st century” points to the need for a global health workforce that is competent to deal with medical issues in a globalised world. They endorse the inclusion of a global health perspective in the existing medical curricula and the provision of distinct courses and training sessions [11]. Nevertheless, the commission also expresses the need for further clarification of the definition, contents and strategy of Global Health.

### Global Health – Myriad of definitions?

**Fig. 1:**
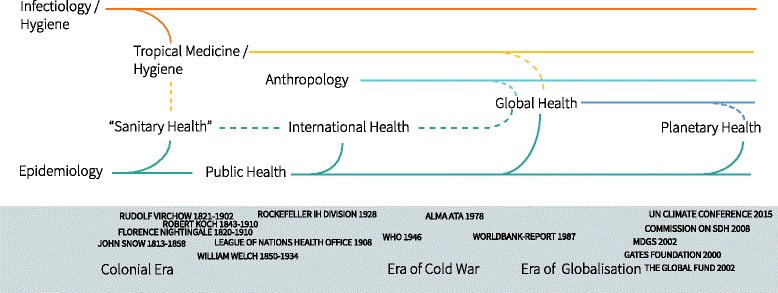
The Origin and Development of the term ‘global health’ in relation to other disciplines and historical events. WHO: World Health Organization; MDGS: Millennium Development Goals; SDH: Social Determinants of Health

Most authors trace the history of Global Health (GH) back to its roots in International Health (IH), Public Health (PH) and Tropical Medicine (TM) [12, 13]. An illustration of these relationships and their historical context is presented in Fig. 1. It shows the multiple relations between GH and other healthcare fields as well as political and social movements.

An overview about suggested definitions of GH can be found in Additional file 1. Most definitions refer to the global or worldwide health status and determinants of health, thereby defining ‘global’ in a sense of interdepending nations [13], as distinct from international [14], as transcending national boundaries [15], as undermining territorial boundaries [16] or ‘global’ in a universal or worldwide [17] sense. Bozorgmehr et al. suggest a dialectic approach to understand global as holistic, as supraterritorial and as worldwide [18]. Further ambiguity exists regarding the entity of GH (as academic discipline [19], a field [20] area [21], a mindset [13]) and regarding a normative objective as part of the definition (e.g. ‘health equity’ or ‘health for all’) [18, 22].

The term global health is used in a variety of settings [23]: many NGOs in the humanitarian and development sector, but also academic institutes and courses use the term GH [24] as do the fields of international relations and global health governance [15, 25, 26]. Stuckler et al. argue that there are at least five metaphors about global health and especially global health policy: as foreign policy, as security, as charity, as investment and as public health [27]. Some authors suspect GH to be just a ‘buzz word’ replacing the term international health [14, 28]. However, most authors argue that the global, supraterritorial aspects of GH, the combination of individual and population health and a strong interdisciplinary approach justify the distinction from Public Health and International Health [18, 21].

The most common definition has been published by Koplan et al.: “global health is an area for study, research, and practice that places a priority on improving health and achieving equity in health for all people worldwide.” [21]. This definition has been affirmed and adopted by the Consortium of Universities for Global Health [29], the Canadian government [17], and (partly) by the German Academy of Sciences [30], even though other definitions are used as well [30, 31].

### Global Health and medical education in Germany

In the context of the G20 summit a report by Kickbusch et al. [31] describes the “low prioritisation of global health in its universities’ curricula” and the – compared to North America and the UK – low level of global health education activities – by numbers, degree options and lack of GH education research.

A recent online survey revealed, that only one third of German medical schools offers some kind of education in GH [32]. Another study demonstrated the high student interest in GH and the need for improved GH education, not least due to changed mobility patterns of medical students and the increase in popularity of international electives [33, 34].

The German medical students association has published a suggestion for GH curricula [35, 36] and the national academy of sciences (Leopoldina) recommends the inclusion of global health components in the curriculum of all health professionals [30]. But to date, no general recommendation or standard exist for Global Health Education in Germany.

### Aims of this study

This study aims to gain a deeper understanding of how GH teachers in medical education understand GH, what content they teach and what didactic means they use. We intend to use these results to inform the further development of GH courses in Germany and beyond.

## Methods

### Methodology

To gain a better understanding of GH from within the medical education community, we chose a Grounded Theory-based approach [37–39]. Grounded Theory allows for new theories to emerge from the data and to let these guide the further research process. Following Strübing’s argumentation [40] we decided to base the further research process on a pragmatist epistemological understanding and the Grounded Theory Method as taught by Corbin and Strauss [41].

As we focus on GH teachers the interview technique was influenced by Meuser und Nagel’s concept of expert interviews [42, 43], being initially explorative but ultimately – in alignment with Grounded Theory – aiming for theory-generation [44].

Through this approach we perceive the GH teachers in a two-fold way [44]: (1) as experts with an explicit expert knowledge about GH and GH education and simultaneously (2) as active participants teaching GH, interacting with students and having the power and opportunities to shape GH education at their universities and beyond.

Not least because some of the experts interviewed are active in GH education research themselves, the interactionist paradigm of pragmatist epistemology allows best to account for the dynamics within our research process.

The overall research process followed an iterative-cyclical design. Starting with an extensive literature review the steps of sampling, data collection and analysis were conducted concurrently and informed each other while shaping and specifying the main research question. Extensive memo-writing accompanied all steps to document the development of core ideas and strategic decisions.

### Sampling

We followed a theoretical sampling approach aiming for purposive, maximum contrast sampling [45]. The starting point for our sampling was a semi-formal network of GH teachers in Germany. From there we extended the range of participants through snow-balling by asking for further recommendations and suggestions for experts with a supposedly different perspective that were teaching at German universities.

During the first interviews the professional, academic and biographic background of the experts was identified as a strong influence of their GH understanding. As repeated reference to student-led initiatives appeared, we also included a student perspective. Aiming for further contrast and working against the risk of interviewing like-minded experts only, we also approached medical professionals who were a) not teaching medical students, b) coming from a non-European / non-western country, c) teaching GH-related topics but choosing intentionally other titles than GH. Inclusion criteria were experience with teaching GH-related aspects and an academic background in medicine.

To ensure anonymity of the experts [46] we can only provide an accumulated list of the diverse set of academic, professional and biographic backgrounds and other participants characteristics (see Table 1).

**Table 1:** Characteristics of Participants

Gender (f/m)	4/7
Age (years)	25–71
Academic Background	All: medicine
	Additionally: Tropical Medicine, Epidemiology, Public Health, Theology, Anthropology, History and Ethics of Medicine, International Health, Ethnology, Medical Anthropology
Biographic and Professional Background	All: Experience of teaching and of living in a foreign country
	Additionally: Official Development Assistance, NGO Senior management, medical work within FBOs, ethnological research in LLDC, medical services for migrants in Germany, Development Aid Consultancy, Political Work, Support of student initiatives, research fellowships, intercultural background of core family, born in LLDC, education research
Duration of interviews	45–120 min
Participation in Member Check	7 of 11

Recruiting and data collection took place over 13 months and was primarily research-guided. End point was the criteria of data saturation as understood by Morse [47].

### Data collection

Interviews were carried out by MH on-site at a location suggested by the interviewees. All interviews were audio-recorded and transcribed verbatim. After each interview the interviewer wrote a memo containing first impressions of the interview noting especially unexpected answers or directions. Most interviews were conducted in German, one interview was conducted in parts in English. For this paper all quotes were translated into English.

The initial interviews followed a semi-structured interview guide covering a range of topics identified by prior literature research, though leaving space for narrative elements and additional aspects from the interviewee. The first four interviews were reviewed by a second researcher (SB) and the interview guide adapted. After the first sets of interviews we increased narrative components and added new aspects from the early analysis process. Interviews took regularly 45–120 min. One interview was conducted as focus-group with two interviewees.

### Analysis

The first steps were the same for all interviews: analysis started by immersion into the transcript and informal noting of first impressions. The next step was careful line by line reading and inductive coding of the interview contents. Where possible, original terms from the interviewee were used. Emerging concepts or abductive flashes [48] were noted in form of memos and evaluated later. Based on the semantics of the first coding process initial categories were formed.

Two researchers (MH and SB) coded several interviews independently. They compared codes and codings afterwards and used differences to further refine their coding approach, following the idea of consensual coding [49]. Later interviews were only spot-checked for traceability by the second researcher (SB).

After conduction and analysis of the first four interviews we analysed all codes independent of their provisory categories: We used an open, semantic card-sorting method [50] to build a code tree. About 400 Codes were printed out and sorted in categories by asking: “What question is answered by this code?” Results were checked for traceability by SB. The emerging four main categories and their subcategories laid the foundation for the coding of the following interviews (see Table 2). This code tree was presented and discussed in an interdisciplinary qualitative research group not affiliated with GH topics to check for clearness and intelligibility.

**Table 2:** Main Categories emerging from initial card sorting after the first four interviews

(1) What is Global Health?	
a. Historical Background	
b. Definition	
c. Distinction from PH/IH/TM	
(2) What belongs to GH?	
a. Meta-Aspects	
b. Overarching Goals	
c. Concrete Aspects	
d. International Electives*	
(3) How can GH be taught?	
a. Target Group	
b. Didactics	
c. Form	
(4) What is important for the future of GH?*	
a. GH Workforce	
b. GH Education in Germany	

Legend: Items marked with an asterisk have not been used for the analysis of the data for this paper

To aid with constant comparisons we used matrices and tables summarising main concepts from the interviews and comparing emerging topics and biographical backgrounds of experts [51].

The following interviews brought up further topics and codes and led – where necessary – to a resorting of aspects within the code tree and introduction of new comparisons within and between the interviews. Throughout the interviews we decided to discard the 4th category (Future of GH) and to only use it to support analysis of the other three categories.

### Participant validation

As several of the interviewees were themselves involved in GH education research and as all provided a high reflective capability we used the concept of participant validation / member check to further our understanding of GH and to reveal misunderstandings or misrepresentations within our analysis. Our approach could be best compared to the method of synthesized member checking [52]:

After finishing data collection with a first set of considerable ‘stable’ results of our analysis we summarized key aspects of our research in text form and illustrations. These were aggregated in form of an online-survey. We asked for overall agreement for each finding on a likert-type scale and for additional free text comments. The survey was sent to all interviewees several months after finishing data collection. Seven out of 11 participants answered our online surveys. Answers were compared to the interview data and current analysis and were used to refine. Due to the small sample size no further statistical analysis was undertaken.

## Results

### Global Health as umbrella term

Throughout the first interviews it became notable that the understanding of GH is closely linked to the biographical background, research focus and interests of the interviewee. When asked about the topics that are considered part of GH, one expert drew this connection by saying: “*Going back to my own biography, I believe, it [Global Health] should cover…*” (I5:11). Another interviewee said: “*When I started thinking about Global Health, I realised that this [access to medicines] and the pharmaceutical industry ran like a common thread through my life. […]”* (I4:16). Going further back, evidence could be found in the first interview already: “*We need to stop thinking medicine in terms of organs and organ systems, we need to start realising again – and I’m a medical historian – […] that we deal with people, with human beings and their backgrounds – social, cultural, societal, biographical […] it contains many facets, but healthcare depends on the context of human life and that is Global Health for me.*”

Asking about the key characteristics of GH, an at first sight surprising aspect appeared. This aspect turned into a key element of our understanding of GH. The expert said: “*I think it [GH] offers the opportunity to integrate topics that I believe are important for university teaching and that have not been covered sufficiently so far under this new title.*” (I5:11). This statement did not align with our presumptions about GH and the way it was concepted for university teaching, but we could actually see how it fitted with the other interviewee’s approaches as well. The same expert explained later: “*Within the Global Health movement I’m not proposing the idea of a strict delineation or even separation [of Global Health and Public Health]. […] To me Global Health is primarily a perspective.*” (I5:43).

Consecutively, we took up this idea of understanding GH as a panoply or collective term and discussed it in the following interviews:

*Expert J: I believe, at the end of the day we cannot avoid understanding Global Health as a panoply that, even though other disciplines might not like it, encompasses Public Health, International Health and aspects of tropical medicine. It’s – so to speak – kind of an ‘umbrella term’. But it has this – let me call it component – this, to put it simply, global or supraterritorial dimension. And this is a unique feature that sets it apart.* (I9:48)

Iterating our analysis with this idea of GH as ‘umbrella term’, we looked how this approach would answer our initial questions. According to Merriam-Webster an umbrella term is “something which covers or embraces a broad range of elements or factors” [53].

**Fig. 2:**
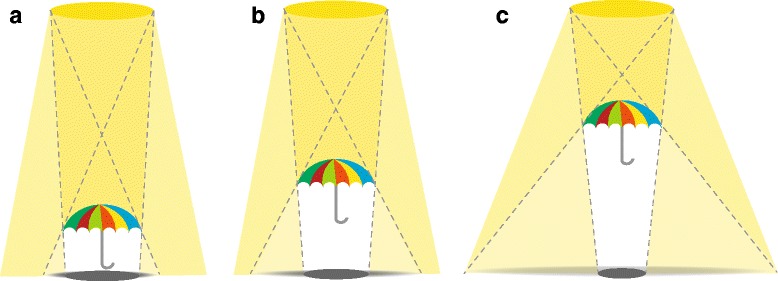
Global Health (GH) as Umbrella Term: The ratio of umbra and penumbra. Legend: the umbrella casts a shadow with an umbra (core shadow) and penumbra. The ratio of umbra and penumbra depends on the distances between light source, umbrella and screen. **a**-**c** Demonstrates this effect by adjusting the umbrella between light source and screen. This can be transferred to GH: a close / narrow definition (**a**) risks to lose important aspects of the penumbra. While a very broad understanding of GH could lead to arbitrariness and irrelevance

Literally, an *umbrella* is a little shield that provides shadow. Depending on the proportions and position of the light source and the screen or projection surface, an umbrella casts a complete shadow in the centre (*umbra*) and a more diffuse semi-shade (*penumbra*) around the centre (Fig. 2b).

The ratio between umbra and penumbra is determined by the distance of the umbrella to the screen. The closer the umbrella to the screen the smaller the portion of the penumbra, until it eventually ends with just the umbra below the umbrella (Fig. 2a). It is very specific but has lost its original function. Taking the other extreme by choosing a long distance between umbrella and screen the umbra decreases and ultimately the penumbra disappears in meaninglessness (Fig. 2c).

This can be applied for GH as well: having a very strict understanding of GH – sharply distinguishing GH from other fields – brings the risk of losing valuable GH aspects that are only covered by the penumbra but that constitute strengths of GH. On the other hand, not defining a genuine GH core and accepting everything under the GH umbrella can lead to arbitrariness and hence turn the field irrelevant.

### What is Global Health?

**Fig. 3:**
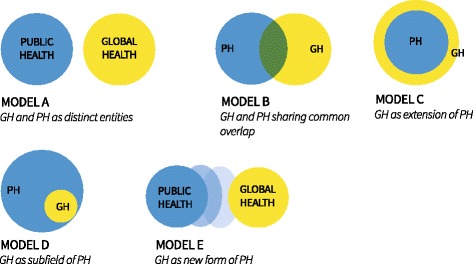
Theoretically possible relationships between Public Health (PH) and Global Health (GH). We use the form of an adapted Venn diagram to describe the theoretically possible relationships between GH and PH. Most experts found model B the most useful even though its interactions are more complex than reflected in this two- dimensional model

Asked about the character of GH, most experts referred to the relationship between GH, PH, IH and TM. Though most claimed differences between GH and the other fields, the actual relationships were described in various, partly contradicting ways. We tried to illustrate the understandings found in the interviews exemplary for GH and PH by drawing two circles and the different ways they relate to each other (Fig. 3).

There was broad agreement among the participants that GH is somehow related to PH and the other disciplines, as one expert described it: “*Global Health cannot be thought or understood without knowing and understanding Public Health as a concept, as an element and as an approach*” (I9:48).

But regarding the concrete relationship we found contradicting statements ranging from “*There is Global Health in every aspect of Public Health*” (I8:25) to *“I do not believe that the competencies needed for Global Health would differ fundamentally from those needed for Public Health in Germany*” (I3:11) to: “*There are certain colleagues, who teach Public Health themselves, who claim Global Health is in fact Global Public Health, but I don’t agree, because from my point of view [Global Health] also considers curative healthcare.*” (I5:43).

We presented the different models to the experts after the interviews in the digital survey and asked them, which one resembled their opinion best (Fig. 3). All of them chose either model B (GH and PH sharing common overlap) or model C (GH as extension of PH), but also used the comment field to express that none of them perfectly resembled their understanding.

One expert in the interviews wondered whether we needed Global Health at all, and whether ‘International Health’ was more meaningful: “*We need the term ‘international’. And I think, this is what students can relate to, because many of our students want to pursue something ‘international’, be it a nursing internship, an elective or part of their residency. And this is not covered by the term ‘Global Health’. Its meaning is not obvious*” (I7:59). Yet, another expert warned not to lose the genuine unique aspects of GH: “*I find it problematic that many Global Health concepts neglect what I would consider the unique aspects of GH. Eventually they just teach International or Public Health stuff or health in other countries -or even just healthcare in other countries.*” (I9:48).

Broad agreement was reached on the statement that GH should not be a distinct discipline or subject, but rather a field or area: “*I believe, Global Health is just not a discrete subject. That would make no sense.*” (I1:30), “*To me, Global Health is essentially a perspective. I would oppose the idea of creating a new subject. […] I really think the US definition that says it’s an interdisciplinary area is way better...*” (I5:43).

**Fig. 4:**
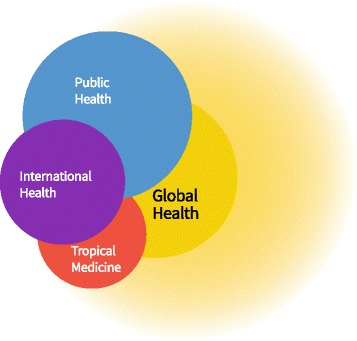
Global Health as Umbrella Term and its relationship to Public Health, International Health and Tropical Medicine. This illustration shows GH with its umbra (core shadow) and penumbra. Its core overlaps with all three disciplines that also overlap with each other. If one draws a very wide penumbra it might encompass most of the aspects of the other disciplines but we would normally assume that each discipline still has its distinct unique areal

Taking the understanding of GH as umbrella term resolves some of the tensions (Fig. 4): GH as an emerging perspective covers the unique and defining features of GH at its core (casting the umbra), but it can also cover further aspects from other fields like PH, IH and tropical medicine (casting the penumbra). PH, IH and TM all share some overlaps between each other. And PH, IH and TM all contribute to core aspects of GH – be it in terms of research methods, competencies or topics covered. But GH also brings its own genuine aspects like the supraterritorial dimension. The ratios of the different items can certainly be debated, but one needs to remember the greater the penumbra the less specific the GH core.

### What belongs to Global Health?

The experts mentioned a variety of topics they taught in their courses and topics that they prioritised or found relevant for GH students. Again, we could show that these topics were often linked to the biographical background of the expert: “*Initially [the topics taught] came from my medical and research experience…*” (I5:39) or “*And then I always teach something about [specific topic], but this is also because it is my hobbyhorse.*” (I6:101).

Another important aspect regarding the choice of topics was brought in by several experts: “*I think there are three target groups that overlap, but that would need different kinds of teaching.*” (I6:35). The groups described by this expert and by others could be summarized as (a) medical students without a special interest in GH, (b) medical students who encounter GH – be it through a special interest or work experiences in other countries / international electives and (c) those medical students that pursue a professional career in this field.

**Fig. 5:**
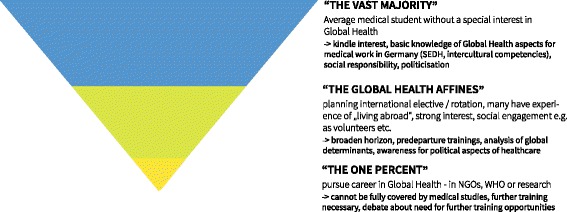
Target Groups of teaching Global Health during medical studies. This funnel represents different target groups among medical students. The size of the sections represents their quantity. The narrower the section the more specific and detailed the teaching needs to be. (SEDH: Socio economic determinants of health; NGO: Non-Governmental Organisation, WHO: World Health Organization)

We used the idea of a funnel or inverted pyramid to represent these groups with their quantity and the specificity needed for their training (Fig. 5). While the “*vast majority*” (I9:66) would need a general overview and basic skills that are of use while working as domestic doctors, “*the 1 %*” (I4:76) would need specific training to foster their professional career in GH. In between there are the “*Global Health affine students*” (I4:76) with an interest in GH, who might consider a GH career, but are primarily interested in broadening their horizons. This group could also encompass candidates for international electives who would need predeparture training.

Asking about the first group, the “vast majority”, we found some agreement on the most important topics: *“… equality, social determinants, human rights – every student should have heard about that, though not every ophthalmologist will later have to deal with it*” (I4:82) or “*There is equity, human rights – but not just declaring them; there is the foundations of healthcare with its systems of financing; there is this intercultural dimension. I believe, these are the three most important things, that can be applied everywhere.*” (I1:53).

**Fig. 6:**
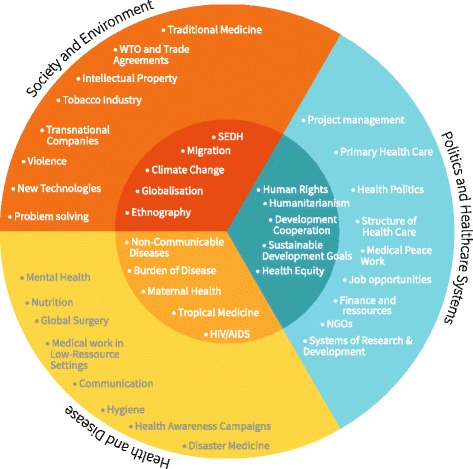
Topics of Global Health by cluster and core versus peripheral aspects. This illustration shows the most common topics for GH that were mentioned in the interviews. They are sorted by inductively formed topical clusters and been assigned to the umbra (core / essential) aspects or the penumbra (extended / peripheral) aspects. The list and assignment to the categories is neither generalisable nor extensive but of exemplary nature. (WTO: World Trade Organization, NGO: Non-Governmental Organisation, SEDH: Socio economic determinants of health)

We analysed the topics mentioned during the interviews and sorted them by three topical clusters and – in a first approximation – by core aspects and extended aspects. These results can be found in Fig. 6, showing the most important and genuine topics in the umbra surrounded by further topics in the penumbra.

### How can Global Health be taught?

Throughout the recruitment process we tried to interview experts from a diverse set of teaching forms that reflect the variance in current GH teaching in Germany: facultative courses, GH lectures within mandatory courses, special focus curricula, excursions, student-led GH initiatives, summer schools etc. A list of mentioned forms can be found in Table 3.

**Table 3:** Forms of GH teaching

Master degree, Massive Open Online Course, Curricular teachings (learning helix), elective courses, Summer school, as part of clinical subjects, special interest tracks / curricula, predeparture trainings, intercalated degree, excursions	

Legend: Forms of GH Teaching as mentioned by the interviewees or used in their taught courses

Most experts favoured a longitudinal approach throughout the whole medical curriculum, allowing for what one expert called a “*learning helix*” (I7:129), including GH aspects in different subjects:

“*I think, Global Health is a topic for the whole curriculum. And it is as cross-sectional – if you want to put it in a radical way – as anatomy or physiology. We need to consider the challenges today’s doctors have to deal with.*” (I1:35) Or another expert said: *“My long-term goal would be to bring global aspects into basically every clinical subject.”* (I5:33).

Next, we asked the experts about the didactic approaches they use in their classes and especially those that they find helpful in teaching GH aspects.

One of the aspects frequently mentioned was inter- or transdisciplinarity: “*We need to teach GH as overarching topic where people are brought together and work together in a multidisciplinary or interdisciplinary way.*” (I4:92).

*“I believe one cannot teach Global Health and one cannot do GH without cultural sciences, history, economy and politics. And you need experts from these fields for that. This cannot be done by a physician who has read a book about it or who is interested in it, but we need economists, law, social or cultural scientisty. They should not just be an add-on to calm our conscience, […], but we need really new research designs and new ways of teaching.”* (I1:32)

Speaking about the differences of trans- inter- and multi-disciplinarity one of the experts explained: *“There are different definitions, but ‘transdisciplinary’ means it also includes the implementation, not just original research.”* (I3:19) Further didactic approaches during the interviews were problem-based learning, transformative education, the promotion of reflexivity and the use of new technologies.

Another aspect mentioned several times was the need for authentic teachers in GH who would bring in their own background and experiences as living examples: “*I think it is really important to encounter authentic representatives of this field, because this is not part of the normal medical studies.*” (I5:69), “*If you bring your own experiences and the teacher tells vividly about them, it will capture everybody’s attention.*” (I8:97). “*They bring a lot of expertise and it is not just something they have read about. It’s all authentic. And that is what we find in evaluations as well.*” (I7:127).

Understanding GH as umbrella term helps to illuminate the goals behind GH, how they relate to each other and how GH can support the overall goal of training physicians for the twenty-first century: At its core we find the goal of understanding supraterritorial determinants of health (Fig. 6). Understanding these leads to politicisation*: “What influences health? Let alone speaking about poverty and its impact on bad health. Let alone speaking about that is actually a political field that you touch automatically.”* (I10:71). Politicisation is mostly understood as the awareness of a political dimension and formation of an opinion: “*I believe, there is a duty to take part in the public discourse about this topic. And to do that, you need to understand the contexts and links that lead to health crises.*” (I3:25). Wondering whether the goal of politicisation would leave neutral grounds, one expert said: “*I want my students to be politicised. I do not want to impinge* ***what*** *they should think, but I want to impinge them so* ***that*** *they think. And that is what I’m doing with my students in class.*” (I4:24). Another expert summarised this goal by saying: *“We want to educate students to be mature citizens in white coats”* (I5:23).

*“I believe, Global Health, if it politicises people, inspires them to think, to think globally, to think complex, and to think about health – and if they are then politicised – it would be sufficient to me.” (I4:64)*

Closely related to politicisation and often interlinked is the goal to encourage responsibility among students: *“These days we need to bring students to the point, that they take on responsibility.”* (I4:22). GH is an opportunity to encourage responsibility and to reflect on values: “*And this is a part, I believe, where we can contribute within in the university, but especially in Global Health, that we have a responsibility to transport different values again.*” (I4:58) GH can serve to encourage engagement not just in other countries but within our own society: “*Many students do not think about supporting socially marginalised or disadvantaged people in Germany and it is not really attractive. They would rather do an elective in Africa than in a homeless shelter. […] I think, this is a very important, central aspect, of what I try to teach the students.*” (I6:35).

GH with its interdisciplinary character and its focus on social and economic determinants is predisposed to include aspects of liberal arts and social sciences into medical education:

“*Exactly, I think, this is a field, where we can foster aspects of social sciences within the medical training. And we can use a topic, that is actually very interesting and that allows us to reach out to students by their call it Albert Schweitzer-motive.*” (I9:34).

To understand the determinants of health we need other disciplines and ways of thinking: *“We can often build a bridge between organic symptoms and social, political, societal and economic aspects. And we see part of that inside the laboratory or with conservative medical procedures, but there is a lot that we do not see and we need to make that visible. And this does not happen by conventional medical thinking alone.”* (I1:32).

Several experts stressed the need for case studies, simulation games, and problem-based learning: “*We need people, who can work together, who can think in new ways, who are creative and who can react to new approaches.*” (I4:62). Some experts using case studies retold the positive impact of these: “*They [students] want to be challenged, want to take part, and that is when teaching works best.*” (I7:135).

**Fig. 7:**
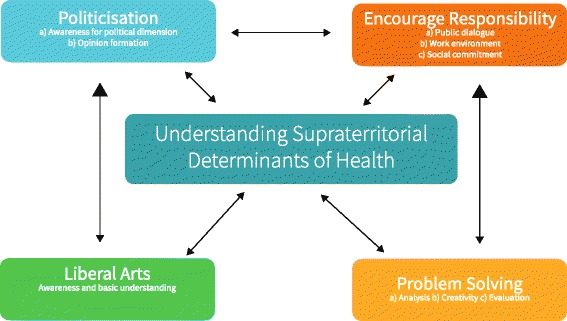
Central Goals of GH teaching. This graphic visualises how the central aspect of understanding supraterritorial determinants of health is reciprocal linked to the surrounding goals of politicisation, encouraging responsibility, an understanding for liberal arts and problem solving

Figure 7 shows the different goals and how they relate to each other and reinforce each other. These goals reveal why GH does not just benefit in itself, but can help improving overall medical education: “*We eventually need to train doctors – and that’s important from my point of view – to deal with the complexity present these days*.” (I1:33) Aspects like interdisciplinarity are not just for GH experts relevant, but also for traditional medical work. *“[Health equity] demonstrates, the didactic potential of Global Health to improve medical education in general.”*

### A new model of Global Health

**Fig. 8:**
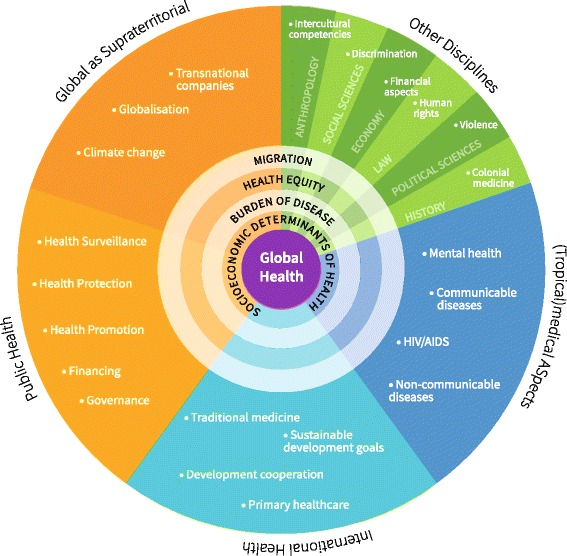
Global Health as Umbrella Term: An interdisciplinary model. This model reflects the understanding of Global Health as umbrella term: it shows how topics from a diverse set of disciplines build the foundation for Global Health. The circles in the centre show how different disciplines relate to each other and supplement each other. They are further unfolded in illustration 9. In this model we have added ‘Global as supraterritorial’ as an own category to underline how it adds to the other fields, even though some of the topics are also covered by other disciplines. All topics used in this illustration are just exemplary

**Fig. 9:**
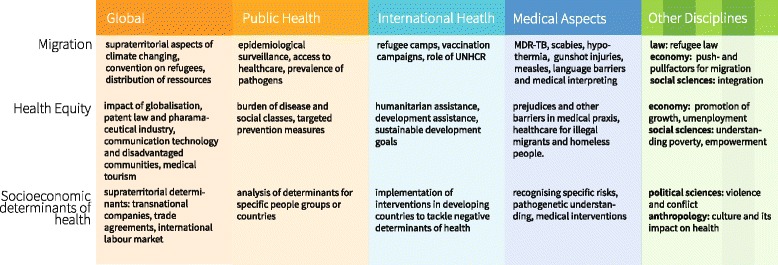
Global Health as Umbrella Term: Unfolding the interdisciplinary character. This illustration uses three of the inner circles from illustration 8 and shows how these topics benefit from an interdisciplinary lens

Understanding Global Health as umbrella term with core aspects (umbra) and extended aspects (penumbra) was the starting point. Taking its interdisciplinary character and its relationship with IH, PH and TM and a variety of topics mentioned during the interviews led to the following new model of GH (Fig. 8): GH comprises aspects of (tropical) medicine, IH, PH and other disciplines. Additionally it includes global aspects in the sense of “global as supraterritorial”. We used some exemplary topics to clarify what could be part of these subjects, though the topics mentioned are neither representative nor exclusive. Surrounding the core we present some typical cross-sectional topics, which are explained in more detail in Fig. 9, revealing how these topics can be integrated by different disciplines.

### Validation survey

We presented the key results of our analysis to the experts interviewed. Participants mostly agreed with the models and statements presented. They used free text comments to state the importance of a non-technocratic, not medically dominated understanding and suggested further topics or aspects that had not been mentioned before. Especially disagreement was used to further refine the models presented in this research.

## Discussion

### Main findings

We conducted in-depth interviews with 11 medical educators from the field of GH. Their answers allow insights into the diversity and variety of GH education in Germany. Understanding GH as an umbrella term helps to account for these differences.

### Characteristics of GH

Our interviews resemble the general discourse in the literature to a far extend: tracing its origin back to TM, PH and IH [12, 21, 54, 55]; debating the relationship from GH and PH [21, 56, 57] and discussing an inherent moral or political stance of GH [18, 22, 58]. Other authors have – similar to the experts in our interviews – argued that GH is actually PH [56], that GH is best reflected in social medicine [59] or that it is a discipline on its own [60], while Koplan et al. [21] described distinct features of GH that set it apart from PH and IH. Bozorgmehr was the first to suggest a dialectic understanding of GH. He argued that the different understandings of GH complement each other [18].

Our study goes beyond mere definitional issues by applying the concept of GH as an umbrella term to GH education in the field of medicine. GH needs to incorporate many disciplines. It benefits from the flexibility and diverse range of possible topics but also needs to meet a minimum standard covered by the core-aspects.

One could wonder whether the concept of GH as an umbrella term is necessary to capture the essence of GH. We argue that this ambiguity captured by our concept is an essential part of globalisation itself and is hence reflected in GH as well. The sociologist Scholte summarises the complexity: “*In sum, current globalisation is not replacing one compact formula (territorialism) with another (globalism). Rather, the rise of supraterritoriality is bringing greater complexity to geography – and by extension to culture, ecology, economics, history, politics and social psychology as well. The relative simplicity of a territorialist-statist-nationalist world is fading.*” (p.1494) [3].

### Content of GH

Our analysis identifies three topical clusters describing the content of GH: (1) health and disease, (2) society and environment, (3) politics and healthcare systems. The decision to list contents / topics and not competencies reflects the wording of most interviewees – even though some experts mentioned the need to define competencies and not just knowledge-based learning outcomes. Johnson et al. [61] proposed a set of six learning outcomes for medical students that align well with our three clusters. The Consortium of Universities for Global Health [29] defined – similar to our finding of the different target groups – different sets of competencies depending on the level of the student. Their basic level ‘global citizen level’ covers 8 domains of competency. Their competency-based approach goes beyond our content model and especially the aspect of ‘professional practice’ has not been explicitly covered by our content model, but is reflected in the didactic’s section on transformative education. An overview about contents for GH can be found in Additional file 2. One could carefully conclude, that contents identified within our study do reflect the broader consensus in literature. It is nevertheless necessary to stress, that this study does not allow to derive a prescriptive listing of GH contents as this was neither the intention of our research nor would the method used allow for generalisability.

### Didactics of GH

We described a number of different forms of GH teaching (Table 3). Many of them can be found as case examples in the literature and are mentioned in the literature review on GH competencies and approaches by Battat [62] as well. The most outstanding is probably the integration of GH in the whole curriculum in form of a “learning helix”.

Touching on some underlying didactic principles we find some that are widely reported (e.g. inter- and transdisciplinarity [11, 63], problem-based-teaching [64]), and others that appeared only recently in the medical education landscape: the idea of transformative education [65]. The Lancet commission Health Professionals for the twenty-first century brought this concept also into medical education [11]. Another aspect worth mentioning is the need for authentic teachers. This is a concept that to our knowledge has not been brought up in medical education so far.

Figure 7 summarises these main aspects of GH around the core of understanding supraterritorial determinants of health. It also shows how these factors support each other and explains how GH can support overall medical education and the formation of a physician’s professional identity [66].

### A model of GH as umbrella term

Our final model (Figs. 8 and 9) summarises these findings and presents the field of GH for medical education as a field with different disciplines contributing. The circles in the centre signify the value of its interdisciplinary character. Bozorgmehr et al. have drafted a semi-circular model of Global Health starting further explaining the territorial domain of GH [18]. While their model explains the territorial and supraterritorial aspects in much more detail, our model focuses on the overall relationship to other disciplines and how this can be applied to medical education.

### Limitations and strengths

This study focuses on the GH understanding of German GH teachers in medical education. Its main limitations are indebted to its specific focus and exploratory Grounded Theory methodology. Our purposive, maximum contrast sampling aims to reflect a broad range of diverse opinions. Major parts of the research process have been triangulated by two researchers and presented in an interdisciplinary qualitative research group. Participant validation helped to minimise misrepresentation of the interviewees. Nevertheless, it does not allow for simple generalisations and needs to be interpreted in rather conceptual than technical terms.

We have tried to include experts with different academic and biographic backgrounds, but we are still aware that our sample does not correspond with the interdisciplinarity and globality demanded for GH education. While it does reflect the reality of the current German medical education landscape, this should not deter future research from looking into more innovative, diverse and participatory approaches that go beyond the medical community in Germany. The understanding of globalisation’s impact on health could differ significantly from other countries and needs further research.

## Conclusion

Understanding GH as an umbrella term, helps to shed light on a number of discussions: the relation between GH and the fields of PH, IH and TM; the need for standardisation of core aspects in GH teaching while maintaining the freedom for variety; the diverse and innovative teaching forms and methods used in GH so far and – by using the inverted funnel of target groups for GH teaching – the different needs for GH teaching.

Although GH has strong roots in traditional health sciences, it is a young, dynamic, relevant and innovative field that can cater to the needs of the future health workforce. This paper identifies two major challenges for the integration of GH in medical education in Germany: (1) the concrete implementation of GH aspects within and beyond the medical curricula and (2) the balancing of GH’s core characteristics with its wider, extended aspects.

While the first might be a political and academic process that needs to happen in due time, the second aspect of fine tuning the distance of the umbrella casting the umbra and penumbra might remain a continuing challenge to be met by every course and program.

## Additional files

Additional file 1: Overview of Definitions of Global Health. This file lists definitions of Global Health identified by our literature research. (PDF 671 kb)

Additional file 2: Synopsis of Learning Outcomes and Competencies for Global Health Education. This table compares different learning outcomes and competencies of recent years to the findings of our study. (PDF 645 kb)
