# Supplementary material for: Global Health as “umbrella term” – a qualitative study among Global Health teachers in German medical education
Source: Global Health. 2018 Mar 27;14:32. doi: 10.1186/s12992-018-0352-y (PMC5870065; doi:10.1186/s12992-018-0352-y)
Supplement: Supplementary file 1 — Overview of Definitions of Global Health. This file lists definitions of Global Health identified by our literature research. (PDF 671 kb) [file 12992_2018_352_MOESM1_ESM.pdf]

Supplement 1: Definitions of Global Health

1991	Jain [1]	Global Health, like any other concept, is first of all a mindset, which is neither rooted in the notions of "Us" and "Them" nor obsessed with international diplomacy, nor engrossed with the priorities and workings of international organizations, but which recognizes the field of health as a field in which interdependence of nations is an endemic and germane characteristic.
2003	Lee [2] (p.191)	Global Health concerns the way in which globalization is impacting on both the determinants of health and health outcomes.
2006	Brown et al [3]	The term “global” is also associated with the growing importance of actors beyond governmental or intergovernmental organizations and agencies—for example, the media, internationally influential foundations, nongovernmental organizations, and transnational corporations. Logically, the terms “international,” “intergovernmental,” and “global” need not be mutually exclusive and in fact can be understood as complementary
2006	Kickbusch [4, 5]	Global Health refers to those health issues that transcend national boundaries and governments and call for actions on the global forces that determine the health of people.
2008	MacFarlane[6]	This new phenomenon seems to reflect: (1) a greater international connectedness […] (2) more public awareness of common vulnerabilities to the spread [diseases, …] (3) discomfort that there are huge inequalities in disease burden […]
2008	The United Kingdom [7]	Global health refers to health issues where the determinants circumvent, undermine or are oblivious to the territorial boundaries of states, and are thus beyond the capacity of individual countries to address through domestic institutions. Global health is focused on people across the whole planet rather than the concerns of particular nations. Global health recognizes that health is determined by problems, issues and concerns that transcend national boundaries.
2009	Janes [8]	It frequently serves as a gloss signalling complexities inherent in linking health and accelerating and intensifying global processes, although it sometimes simply refers to work that has an international (read: low-income country) dimension.
2009	Koplan [9] supported by Canadian Commission [10] in 2012	Global health is an area of study, research, and practice that places a priority on improving health and achieving equity in health for all people worldwide. Global health emphasizes transnational health issues, determinants, and solutions; involves many disciplines within and beyond the health sciences and promotes interdisciplinary collaboration; and is a synthesis of population-based prevention with individual-level clinical care.
2009	US Institute of Medicine [11]	Global health is the goal of improving health for all people in all nations by promoting wellness and eliminating avoidable disease, disability, and death. It can be attained by combining population-

		based health promotion and disease prevention measures with individual-level clinical care.
2010	Beaglehole and Bonita[12]	Collaborative trans-national research and action for promoting health for all
2010	Bozorgmehr [13]	The field is about building and re-building, researching and analysing, teaching and learning the links between social determinants of people's health anywhere in the world.
2010	Lancet Commission Health professionals for a new century, Frenk et al [14]	Consensus is growing about its key tenets—universalism, global perspectives in discovery and translation, inclusion of broad determinants of health, interdisciplinary approaches, and comprehensive framework.
2010	Rowson et al in Bozorgmehr[13]	A field of practice, research and education focussed on health and the social, economic, political and cultural forces that shape it across the world. The discipline has an historical association with the distinct needs of developing countries but it is also concerned with health-related issues that transcend national boundaries and the differential impacts of globalisation. It is a cross-disciplinary field, blending perspectives from the natural and social sciences to understand the social relationships, biological processes and technologies that contribute to the improvement of health worldwide.
2011	Bruchhausen et al (our translation into English)[15]	The focus of Global Health is on health and its social, economic, political and cultural determinants that have a worldwide – in industrialised as well as in developing countries – impact. Simultaneously it analyses diverse global inter-relations that lead to events in one part of the world affecting the health of people in other parts of the world.
2011	Velji & Bryant [16]	For the authors, the concept of global health reaches beyond the rich-poor dichotomy and geographic boundaries and borders to the forces that separate the powerful, free, privileged populations from the population that is powerless and unfree.
2012	European Academic Global Health Alliance [17]	Global health is an emerging field encompassing research, policy and practice. The concept implies an understanding of the interdependence and complexity of influences on human health.
2012	The European Commission[10]	It is about worldwide improvement of health, reduction of disparities, and protection against global health threats.
2013	Frenk [18]	Global health should be defined by two key elements: its level of analysis, which involves the entire population of the world, and the relationships of interdependence that bind together the units of social organization that make up the global population (e.g., nation states, private organizations, ethnic groups, and civil society movements).
2014	Chen [19]	A fundamental task of global health science is to understand the aetiology of medical and health conditions with a global perspective and to obtain concrete data supporting intervention strategies to improve health.
2015	Liu, Yan [20]	Global health implies consideration of the health needs of the people of the whole planet above the concerns of particular nations [...] Global health education reflects the increasing attention to social accountability in medical education.

References

1. Jain SC: **Global health: emerging frontier of international health**. *Asia Pac J Public Health* 1991, **5**(2):112-114.
2. Lee K: **Globalization and Health: An Introduction**: Palgrave Macmillan UK; 2003.
3. Brown TM, Cueto M, Fee E: **The World Health Organization and the transition from "international" to "global" public health**. *American journal of public health* 2006, **96**(1):62–72.
4. Kickbusch I, Lister G: **European perspectives on global health: a policy glossary**. European Foundation Centre; 2006.
5. Kickbusch I: **The need for a European strategy on global health**. *Scand J Public Health* 2006, **34**(6):561-565.
6. Macfarlane SB, Jacobs M, Kaaya EE: **In the name of global health: trends in academic institutions**. *Journal of public health policy* 2008, **29**(4):383–401.
7. HM Government: **Health is Global: A UK Government Strategy 2008-13**. In.: Ministry of Health, UK; 2008.
8. Janes CR, Corbett KK: **Anthropology and Global Health**. *Annual Review of Anthropology* 2009, **38**(1):167–183.
9. Koplan JP, Bond TC, Merson MH, Reddy KS, Rodriguez MH, Sewankambo NK, Wasserheit JN: **Towards a common definition of global health**. *The Lancet* 2009, **373**(9679):1993–1995.
10. Campbell RM, Pleic M, Connolly H: **The importance of a common global health definition: How Canada's definition influences its strategic direction in global health**. *J Glob Health* 2012, **2**(1):010301.
11. Committee on the U.S. Commitment to Global Health, Board on Global Health.: **The U.S. Commitment to Global Health: Recommendations for the Public and Private Sectors**. Institute of Medicine. Washington, D.C.: National Academic Press; 2009.
12. Beaglehole R, Bonita R: **What is global health?** *Glob Health Action* 2010, **3**.
13. Bozorgmehr K: **Rethinking the 'global' in global health: a dialectic approach**. *Globalization and health* 2010, **6**:19.
14. Frenk J, Chen L, Bhutta ZA, Cohen J, Crisp N, Evans T, Fineberg H, Garcia P, Ke Y, Kelley P *et al*: **Health professionals for a new century: transforming education to strengthen health systems in an interdependent world**. *The Lancet* 2010, **376**(9756):1923–1958.
15. Bruchhausen W, Tinnemann P: **Global Health in der Medizinischen Ausbildung**. *Deutsches Ärzteblatt* 2011, **108**(42):2223-2224.
16. Velji A, Bryant JH: **Global health: evolving meanings**. *Infect Dis Clin North Am* 2011, **25**(2):299-309.
17. **Global Health Education Declaration 1.0** [www.eagha.org]
18. Frenk J, Moon S: **Governance challenges in global health**. *N Engl J Med* 2013, **368**(10):936-942.
19. Chen X: **Understanding the development and perception of global health for more effective student education**. *The Yale journal of biology and medicine* 2014, **87**(3):231–240.
20. Liu Y, Zhang Y, Liu Z, Wang J: **Gaps in studies of global health education: an empirical literature review**. *Global Health Action* 2015, **8**(1):25709.
