# Supplementary material for: Global Health as “umbrella term” – a qualitative study among Global Health teachers in German medical education
Source: Global Health. 2018 Mar 27;14:32. doi: 10.1186/s12992-018-0352-y (PMC5870065; doi:10.1186/s12992-018-0352-y)
Supplement: Supplementary file 2 — Synopsis of Learning Outcomes and Competencies for Global Health Education. This table compares different learning outcomes and competencies of recent years to the findings of our study. (PDF 645 kb) [file 12992_2018_352_MOESM2_ESM.pdf]

Synopsis of Learning Outcomes and Competencies for Global Health Education

Johnson et al[1]	Bozorgmehr et al[2]	Rowson [3]	BVMD[4]	CUGH [5]	Houpt[6]	Our study
- Global Burden of Disease<br>- Socioeconomic and environmental determinants of health<br>- Health Systems<br>- Global Health Governance<br>- Human rights and ethics<br>- Cultural diversity and health	- Embrace health determinants from the territorial up to the supraterritorial dimension.<br>- Link these dimensions 'adequately' and provide an understanding of their interrelations.<br>- Lead to the literacy and ability of the health workforce to link and transfer local health issues to global contexts (and vice versa).<br>- Facilitate the identification of actions – aimed at the different dimensions – to achieve health equity and health for all.	- International elective exchanges<br>- The effects of poverty and inequality on health<br>- International comparison of health systems<br>- Globalization and health<br>- International comparison of burden of disease<br>- Tropical medicine<br>- Travel medicine<br>- International health and development<br>- International movement of people	- Politics, Policies and Polity<br>- Individual and Population Health<br>- Social and Transborder Determinants of Health	- Global Burden of Disease<br>- Globalization of Health and Health Care<br>- Social and Environmental Determinants of Health<br>- Capacity Strengthening*<br>- Collaboration, Partnering and Communication<br>- Ethics<br>- Professional Practice<br>- Health Equity and Social justice<br>- Program Management*<br>- Sociocultural and Political Awareness<br>- Strategical Analysis*	- Burden of global disease<br>- Traveler's medicine<br>- Immigrant Health	- Health and Disease<br>- Society and Environment<br>- Politics and Healthcare Systems

* Domains marked with an asterisk were only described for ‚basic operational program-oriented level', not for every medical student.

BVMD: Bundesvertretung der Medizinstudierenden in Deutschland (Association of Medical Students in Germany)
CUGH: Consortium of Universites for Global Health

1. Johnson O, Bailey SL, Willott C, Crocker-Buque T, Jessop V, Birch M, Ward H, Yudkin JS: **Global health learning outcomes for medical students in the UK**. *Lancet* 2012, **379**(9831):2033–2035.
2. Bozorgmehr K, Saint VA, Tinnemann P: **The 'global health' education framework: a conceptual guide for monitoring, evaluation and practice**. *Globalization and health* 2011, **7**:8.
3. Rowson M, Smith A, Hughes R, Johnson O, Maini A, Martin S, Martineau F, Miranda JJ, Pollit V, Wake R *et al*: **The evolution of global health teaching in undergraduate medical curricula**. 2012.
4. Bozorgmehr K, Last K, Müller A, Schubert K: **Lehre am Puls der Zeit - Global Health in der Medizinischen Ausbildung: Positionen, Lernziele und methodische Empfehlungen**. *GMS Zeitschrift für medizinische Ausbildung* 2009, **26**(2).
5. Jogerst K, Callender B, Adams V, Evert J, Fields E, Hall T, Olsen J, Rowthorn V, Rudy S, Shen J *et al*: **Identifying interprofessional global health competencies for 21st-century health professionals**. *Annals of global health* 2015, **81**(2):239–247.
6. Houpt ER, Pearson RD, Hall T: **Three Domains of Competency in Global Health Education: Recommendations for All Medical Students**. *Acad Med* 2007, **82**(3):222-225.
